# The Effectiveness of Mindfulness-Based Cognitive Group Therapy on Marital Satisfaction and General Health in Woman With Infertility

**DOI:** 10.5539/gjhs.v8n3p230

**Published:** 2015-08-06

**Authors:** Najmeh Abedi Shargh, Nour Mohammad Bakhshani, Mohammad Davoud Mohebbi, Khadije Mahmudian, Masood Ahovan, Mojgan Mokhtari, Alireza Gangali

**Affiliations:** 1 Health Promotion Research Center, Zahedan University of Medical Sciences, Zahedan, Iran; 2 Children and Adolescents Research Center, Zahedan University of Medical Sciences, Zahedan, Iran; 3 Department of Psychiatry, Zahedan University of Medical Sciences, Bahran Psychiatry Teaching Hospital, Zahedan, Iran; 4 Departmant of Counseling, Faculty of Psychology and Education, University of Allameh Tabatabai, Tehran, Iran; 5 Deptartment of Obstetrics and Gynecology, Faculty of Medicine, Zahedan University of Medical Sciences, Zahedan, Iran

**Keywords:** mindfulness, infertility, marital satisfaction, general health

## Abstract

Infertility affects around 80 million people around the world and it has been estimated that psychological problems in infertile couples is within the range of 25-60%. The purpose of this study was to determine the effectiveness of Mindfulness-based cognitive group therapy on consciousness regarding marital satisfaction and general health in woman with infertility. Recent work is a clinical trial with a pre/posttest plan for control group. Covering 60 women who were selected by in access method and arranged randomly in interference (30) and control (30) groups. Before and after implementation of independent variable, all subjects were measured in both groups using Enrich questionnaire and marital satisfaction questionnaire. Results of covariance analysis of posttest, after controlling the scores of pretest illustrated the meaningful difference of marital satisfaction and mental health scores in interference and control groups after treatment and the fact that MBCT treatment in infertile women revealed that this method has an appropriate contribution to improvement of marital satisfaction and mental health. Necessary trainings for infertile people through consultation services can improve their mental health and marital satisfaction and significantly help reducing infertile couples’ problems.

## 1. Introduction

De Brardis (2014) in his studies entitled “Psychopathology, emotional aspects and psychological counseling in infertility: A review” demonstrated that infertility affects around 80 million people around the world and it has been estimated that psychological problems in infertile couples is within the range of 25-60%. Depression and anxiety in infertile couples is considerably higher than fertile ones and the mental effect of this issue is higher in females compared to males. Psychological consultation can be a useful aid. A study performed by Tao (2012) revealed that male-based infertility has no negative contribution to marital factors. Moreover, infertile male participants expressed higher marital satisfaction from their wives, while females feel their marital relationship less stable and these factors include sexual satisfaction, age of couple, educational level and life quality. Results of various studies demonstrates that mental factors such as depression or anxiety can be a thread for outcome of treating infertility and By improving mental health, psychological treatments together with infertility treatment o effectiveness of infertility treatments, encourage the infertile person to follow up the treatment as well (Shahrestani et al., 2012). Treatment recognition is defined based on the consciousness of someone regarding treatment process whose emphasis is over actively attending emotions and instantaneous thoughts of the patient without judging or evaluating that thought or feeling. After attention accompanied with consciousness, patient enters a strengthening situation made by him/herself and this trend finally leads to matching and improvement of thoughts and totally the quality of life (Younesi et al., 2008). In a study performed by Shahrestani et al. (2012) entitled effectiveness of group cognition therapy based on the consciousness in improvement of perceived stress dimensions and illogical cognitions in infertile women under IVF treatment, results demonstrated that cognition therapy based on consciousness is effective in improvement of illogical cognitions of parents and perceived stress of infertile women under IVF treatment. Results of Fili et al. (2012) corresponding to comparison of effectiveness of behavioral cognitions therapy and cognition therapy based on consciousness, revealed that by treatment methods can reduce rumination of infertile women. In a research performed by Galhardo et al. (2013), results demonstrated that women participating in Mindfulness plans based on plans until its end, showed a meaningful decrease in depression, exterior and interior shame, being trapped and failure. Furthermore, they had a considerable statistical improvement in concentration skills and self-efficiency in confronting infertility, in control group, there was no significant changes in psychological action except reduction of self-judgment. The purpose of this study was to the effectiveness of Mindfulness-based cognitive group therapy on Mindfulness regarding marital satisfaction and general health in woman with infertility.

## 2. Materials and Methods

Recent work is a clinical trial with a pre/posttest plan for control group. Studied community included all infertile women visiting female clinic of Ali Abitaleb hospital of Zahedan city in 2014 covering 60 women who were selected by in access method and arranged randomly in interference (30) and control (30) groups. Criteria for entering the study are as follows: recognition of infertility by obstetricians, minimum one year of recognition, being within the range of 20-45 years, minimum educational level of fifth grade of elementary school. Criteria for leaving the study were as follows: pregnancy before infertility recognition and psychological or drug treatments for mental or physical problem other than infertility. For interference group, the method of recognition therapy based on Mindfulness is applied according to the method described in reference book (Crane, 2009).

### 2.1 The Contents of an 8-Session MBCT Program According to Crane's Book

#### 2.1.1 First Week: Automatic Pilot

Activities per session;

Mindful eating of a raisin;

Body scan meditation.

#### 2.1.2 Second Week: Removing Barriers

Treatment with body scan meditation, ten minutes of breathing with mindfulness and meditation.

#### 2.1.3 Third Week: Mindfulness with Breathing (Body Movements Using This Technique)

Conscious movement;

“Stretching and breathing exercises”, maintaining the expansion of thoughts and the mind through following meditative practices and concentrating on conscious breathing and body parts. This practice might start with a short-time mindful thought, such as “seeing” or “listening”.

#### 2.1.4 Fourth Week: Staying in the Present Moment

5 minutes of seeing/listening to mindful-based cognitive method (awareness of breathing, body parts, sounds, thoughts and informed choices);

3 minutes of breathing- presenting patterned practices to be applied when experiencing uneasy feelings;

Mindful walking.

#### 2.1.5 Fifth Week: Acceptance and Allowance

Meditation meetings with awareness of breathing and body parts. Putting emphasis on how to react to whatever we think and feel, and whatever originates from physical feelings. Creating a difficult condition for practice and discovering the effects of practices on mind and body;

3 minutes of breathing.

#### 2.1.6 Sixth Week: Thoughts are not Real

Meditation meetings- Awareness of breathing and body- presenting patient problems during the practice, and discovering the effects of the practice on body and mind;

3 minutes of breathing.

#### 2.1.7 Seventh Week: How Can We Best Take Care of Ourselves

Meditation meetings- Awareness of breathing, body parts, sounds, thoughts and emotions;

3 minutes of breathing, presenting a problem during the practice and discovering its effect on body and mind.

#### 2.1.8 Eighth Week: How to Use These Points in Future Decisions

Body scan meditation, end of treatment.

In this period, no psychological treatment was applied for control group. Before and after implementation of independent variable, all subjects were measured in both groups using Enrich questionnaire.

Marital satisfaction questionnaire (Olson et al., 1989) included 35 articles and 4 micro-scales of marital satisfaction, communications, conflict resolution and ideal deviation. In the study of Olson, Alpha coefficient of the questionnaire for micro-scales of marital satisfaction, communications, conflict resolution and ideal deviation is 0.74, 0.78, 0.61 and 0.80, respectively. The validity level of the tool through retest for micro-scales was 0.86, 0.81, 0.90 and 0.92, respectively.

General health questionnaire (GHQ-28): this questionnaire includes four micro-scales of body complaint, anxiety, depression and social malfunction. Yaghoubi (1995) reported the sensitivity of the test as much as 0.86 and its characteristic as 0.82. This work also reports the general validity of the tests as much as 0.88 and validity of micro-scales within the range of 0.50-0.81.9 (Oraki et al., 2012).

## 3. Results

In this section, demographic characteristics are described using the frequency and percentage indices. The subjects’ demographic characteristics are shown in table 1.

**Table 1:** The frequency distribution of infertile women according to some demographic features

Variable	Range	Frequency	Percent
Age (year)	20-28	15	25
	29-37	38	63.33
	38-45	7	11.66

Duration of infertility(year)	1-3	12	20
	4-7	42	70
	8-10	6	10

Education level	Elementary	32	53.33
	Secondary	13	21.66
	Diploma	10	16.66
	Bachelor's degree	5	8.33

As seen in Table 1, the age range of the samples was as follows: 15 (25%) were 20-28, 38 (63.33%) were 29-37, and 7 (11.66%) were 38-45. In terms of the duration of infertility, 12 (20%) have been infertile for 1-3 years, 42 (70%) for 4-7 years, and 6 (10%) for 8-10 years. The highest percentage of the duration of infertility was therefore 4-7 years. In terms of education level, more than half of the participants (53.33%) held a fifth grade elementary school degree.

Table 2 shows the moderate averages; that is, the effect of random variable is eliminated statistically.

**Table 2:** Estimated Marginal Means group (Dependent Variable: posttest)

	*group*	*Mean*	*Std. Error*	*95% Confidence Interval*	

				*Lower Bound*	*Upper Bound*
Marital satisfaction	Interference	1.154E2^a^	2.762	109.833	120.898
	Control	1.070E2^a^	2.762	101.423	112.488

Mental health	Interference	55.094^b^	2.116	50.856	59.331
	Control	45.306^b^	2.116	41.069	49.544

a Covariates appearing in the model are evaluated at the following values: Marital satisfaction = 97.6833.

b Covariates appearing in the model are evaluated at the following values: Mental health = 41.6500.

Average of interference group for the variable of marital satisfaction is as much as 1.154 while this is 1.07 for control group which shows the improvement of marital satisfaction of interference group after treatment. Average of moderated scores of mental health in control group and interference group was 55.09 and 45.306, respectively which demonstrates the improvement of average scores in interference group after treatment.

Results of investigation of statistical presumptions revealed that both presumptions of equality of variances and normality are applicable (p<0.05). Results of covariance analysis of posttest, after controlling the scores of pretest illustrated the meaningful difference of marital satisfaction and mental health scores in interference and control groups after treatment and the fact that women have higher marital satisfaction and mental health after applying independent variable (Table 3).

**Table 3:** Summary of results from ANCOVA of variance related to score Mean of marital satisfaction and mental health post test

	source	Sum of Squares	df	Mean Square	F	Sig	Partial Eta Squared
Marital satisfaction	Pretest	5658.905	1	5658.905	22.885	.000	.286
	group	1060.956	1	1060.956	4.291	.043	.070
Mental health	Pretest	4731.948	1	4731.948	35.224	.000	.382
	group	1436.348	1	1436.348	10.692	.002	.158

## 4. Discussion and Conclusion

Results of MBCT treatment in infertile women revealed that this method has an appropriate contribution to improvement of marital satisfaction which was compatible with similar works. Stress of infertility is correlated to the reduction of marital satisfaction (Gana et al., 2014; Rockliff et al., 2014; Mosalanejad et al., 2014; Cserepes et al., 2013; Tao et al., 2012). Mindfulness is sometimes related to romantic relations (Barnes et al., 2007). Furthermore, infertility consultation improves sexual and marital satisfaction in infertile couples (Vizheh et al., 2013). There is a positive relationship between Mindfulness and marital satisfaction and Mindfulness skills may lead to marital satisfaction (OmidBeiki et al., 2014). Carson et al. (2004) showed that Mindfulness treatment improves marital satisfaction. It must be noted that this compatibility may be the result of MBCT treatment characteristics since with regard to MBCT, it is always implied that this strategy is appropriate for those who are depressed, anxious or are not martially satisfied. In study of the second hypothesis, research findings revealed that women treated by MBCT, have meaningfully different mental health from those who are not treated by this treatment method. This means that MCBT treatment is effective in improving mental health of infertile women of Zahedan. This results are in agreement with that of Galhardo et al. (2013), Shahrestani et al. (2012), and Fili et al. (2012). Infertility is one of the factors which lead to lower mental health (Hasanpour et al., 2014; Baghianimoghadam et al. 2013; Ahmadi Forooshany et al., 2012; Peterson et al., 2003). Sherratt et al., (2013) showed that MBCT treatment is effective in reducing infertility-related psychological problems and will result in higher wellbeing scores and psychological distress. Research shows that Mindfulness leads to improvement of mental health (Pots et al., 2014; Tran et al., 2014; Caldwell et al., 2014). In explaining the reason of the effectiveness of MCBT in improving psychological health of infertile women, it can be said that one of the factors resulting in reduction of mental health of infertile women, is daily life stresses and social pressures. Therefore, it seems that MBCT can help people recognize situations which lead to stress and sadness and apply MBCT strategies to confront them. Another explanation for the effectiveness of the aforesaid interference is mental support which leads to the change of vision of infertile women toward themselves. They no longer feel loneliness nor consider their problem as unique and get an environment in which they are able to address their feelings and problems.

Researches prove the mental inconveniences such as anxiety, depression and mental disturbances. Necessary trainings for infertile people through consultation services can improve their mental health and marital satisfaction and significantly help reducing infertile couples’ problems. In fact, the MBCT exercises seem to be effective in the cognitive system and information processing as people become more aware of the present time through techniques such as paying attention to breathing and body and concentrating on here and now. A widespread application of this technique is therefore recommended, given that this type of education is effective and this technique is beneficial in the considered domains. Based on the findings of the present study and considering the effectiveness of MBCT training, a continuous MBCT usage can be suggested to improve the participants’ life and reduce their problems.

Marital satisfaction is a variable influenced by the characteristics of both men and women. Due to men's lack of cooperation and sometimes not being accompanied by women, only the female sample was used. It is suggested that their husbands also be studied by coordinating the families better in future studies. Some other studies are also suggested to be performed on samples with different types of infertility dividing them according to their gender, and study the effect of demographic variables such as age, education, income, and job on infertility levels and types of problems caused by it.
